# Predicting Presynaptic and Postsynaptic Neurotoxins by Developing Feature Selection Technique

**DOI:** 10.1155/2017/3267325

**Published:** 2017-02-12

**Authors:** Hua Tang, Yunchun Yang, Chunmei Zhang, Rong Chen, Po Huang, Chenggang Duan, Ping Zou

**Affiliations:** ^1^Department of Pathophysiology, Southwest Medical University, Luzhou 646000, China; ^2^Department of Anesthesiology, The Affiliated Traditional Chinese Medical Hospital of Southwest Medical University, Luzhou 646000, China

## Abstract

Presynaptic and postsynaptic neurotoxins are proteins which act at the presynaptic and postsynaptic membrane. Correctly predicting presynaptic and postsynaptic neurotoxins will provide important clues for drug-target discovery and drug design. In this study, we developed a theoretical method to discriminate presynaptic neurotoxins from postsynaptic neurotoxins. A strict and objective benchmark dataset was constructed to train and test our proposed model. The dipeptide composition was used to formulate neurotoxin samples. The analysis of variance (ANOVA) was proposed to find out the optimal feature set which can produce the maximum accuracy. In the jackknife cross-validation test, the overall accuracy of 94.9% was achieved. We believe that the proposed model will provide important information to study neurotoxins.

## 1. Introduction

Neurotoxins act typically against channels to block or enhance synaptic transmission. According to the mechanism of action, neurotoxins can be classified as presynaptic type and postsynaptic type [1]. The function of presynaptic neurotoxins is to act at the presynaptic membrane [2]. They usually block neuromuscular transmission and inhibit the neurotransmitter release due to their specific enzymatic activities [3]. Postsynaptic neurotoxins can bind to the postsynaptic membrane and acetylcholine receptors [4]. Thus, the study of presynaptic and postsynaptic neurotoxin will give us important clues for drug-target discovery and drug design.

The function and structure of neurotoxins can be correctly measured by biochemical experiments; however, it is time-consuming and costly. The availability of huge amounts of proteins generated in postgenomic age provides us with an important opportunity to design various computational methods for timely and precisely predicting protein functions. Thus, it is important to develop machine learning approach to predict presynaptic and postsynaptic neurotoxins. Recently, Yang and Li developed an increment of diversity-based method to identify presynaptic neurotoxin and postsynaptic neurotoxin [5]. The benchmark dataset including 78 presynaptic neurotoxins and 69 postsynaptic neurotoxins was downloaded from Animal Toxin Database (ATDB) [6]. The overall accuracy was 90.39% in jackknife cross-validation, which is far from satisfactory. Subsequently, Song proposed using bilayer support vector machine (SVM) to improve prediction accuracy based on a new benchmark dataset [7]. Although the overall accuracy was dramatically improved, the sequence identity of the dataset was so high that the results were overestimated.

To overcome the shortcoming mentioned above, in this study, we developed a new method based on feature selection technique to predict presynaptic neurotoxins and postsynaptic neurotoxins. In the following, we will introduce how to construct a new benchmark dataset, to formulate neurotoxin samples using peptide sequences, and to obtain the expected result produced by best feature subset.

## 2. Materials and Methods

### 2.1. Benchmark Dataset Construction

A high quality benchmark dataset is the fundamental for building a reliable and accuracy model. The Universal Protein Resource (UniProt) provides the scientific community with a single, centralized, authoritative resource for protein sequences and functional information [8]. Thus, we downloaded presynaptic and postsynaptic neurotoxins from the UniProt. Ambiguous information can reduce the quality of benchmark dataset which makes the prediction model unreliable. Thus, we must exclude the protein sequence which contains ambiguous residues (such as “X,” “B,” and “Z”) and which is the fragment of other proteins. High similar sequences in benchmark dataset will bring about overestimation of results. Thus, the CD-HIT program was used to remove the highly similar sequences by setting the cutoff of sequence identity as 80% [9]. According to above screening procedure, the final benchmark dataset included 256 neurotoxin samples which can be formulated as

(1) $$\begin{array}{c} S=S_{Pre}\cup S_{Pro}, \end{array}$$

where the subset *S*_Pre_ contains 91 presynaptic neurotoxins and *S*_Pro_ contains 165 postsynaptic neurotoxins.

### 2.2. The Dipeptide Composition

One of the most important steps in the prediction problem is to formulate neurotoxin sequences with an effective mathematical expression. Generally, we may formulate a neurotoxin by its entire residue sequence as follows:

(2) $$\begin{array}{c} P=R_{1}R_{2}R_{3}R_{4}\cdots R_{L}, \end{array}$$

where *R* denotes the residue of neurotoxin **P** and the subscript *L* is the number of residues of the neurotoxin **P**. We may use some straightforward and intuitive tools, such as BLAST or FASTA, to find the similar sequences. However, these tools are only suitable for the query sequences which have high similar sequences in searching dataset. If there are no similar sequences in the training dataset, they cannot work well.

Machine learning approach can overcome such problem and correctly identify presynaptic and postsynaptic neurotoxins. Thus, we must convert neurotoxin sequences into discrete vector. A simplest method used to represent a neurotoxin is its residue composition containing a 20-dimension vector. However, the sequence order information would be completely lost and hence limit the prediction quality [10–13]. Thus, the dipeptide composition was used in this study. Accordingly, each neurotoxin sample in our benchmark dataset can be expressed as a 400-dimension vector and formulated as

(3) $$\begin{array}{c} P={\left[\;x_{1}\cdots x_{u}\cdots x_{400}\;\right]}^{T}, \end{array}$$

where *x*_*u*_  (*u* = 1,2,…, 400) is the occurrence frequency of *u*th dipeptide and given by

(4) $$\begin{array}{c} x_{u}=\left\{\begin{array}{cc} f\left(AA\right) & \text{when }u=1\\ \;\vdots \; & \;\vdots \;\\ f\left(CA\right) & \text{when }u=21\\ \;\vdots & \;\vdots \;\\ f\left(YY\right) & \text{when }u=400, \end{array}\right. \end{array}$$

where *A*, *C*,…, *W*, *Y* are the single letter codes of 20 native amino acids, respectively. *x*_*u*_ can be calculated by

(5) $$\begin{array}{c} x_{u}=\frac{n_{u}}{\sum_{u}n_{u}}, \end{array}$$

where *n*_*u*_ denotes the number of the *u*th dipeptides in the neurotoxin **P**.

### 2.3. Support Vector Machine

SVM is a very popular machine learning method and has been widely used in bioinformatics [7, 14–18]. The basic idea of SVM is to transform the input vector into a high-dimension Hilbert space and to determine a separating hyperplane in this space. In this study, we used the LibSVM package 3.18 (http://www.csie.ntu.edu.tw/~cjlin/libsvm/) to implement SVM. Because it is more suitable for nonlinear classification, the radial basis function (RBF) defined as $K\left(\vec{p_{i}},\vec{p_{j}}\right)=\exp\left({-\gamma \left\|\vec{p_{i}}-\vec{p_{j}}\right\|}^{2}\right)$ was used as kernel function. In the SVM model construction, a grid search strategy with cross-validation test was used to optimize the regularization parameter *C* and kernel parameter *γ* as the following standard:

(6) 2−5<C<215with  step  of  2,2−15<γ<215with  step  of  2−1.

### 2.4. Performance Evaluation

In this study, we used jackknife cross-validation to test the prediction. In the jackknife cross-validation test, each protein sample in the dataset is in turn singled out as an independent test sample and all the rule parameters are calculated based on the remaining proteins without including the one being identified. The performance of our proposed method was estimated by the following three indexes called sensitivity (Sn), specificity (Sp), and overall accuracy (Acc) which can be expressed as

(7) Sn=1−NProPreNPre0≤Sn≤1,Sp=1−NPreProNPro0≤Sp≤1,Acc=1−NProPre+NPreProNPre+NPro0≤Acc≤1,

where *N*^Pre^ and *N*^Pro^ are the total number of the presynaptic neurotoxins and postsynaptic neurotoxins. *N*_Pro_^Pre^ is the number of the presynaptic neurotoxins incorrectly predicted as the postsynaptic neurotoxins and *N*_Pre_^Pro^ is the number of the postsynaptic neurotoxins incorrectly predicted as presynaptic neurotoxins.

## 3. Results and Discussion

Many published papers have demonstrated that the optimized features could improve predictive accuracy [19–25]. For high-dimension data, some features are noise or redundant information which has negative contribution to the prediction. Thus, it is very important to develop a feature selection technique to exclude the garbage information. The current study will introduce a new feature selection technique based on the principle of analysis of variance (ANOVA).

Two parameters of feature *u* can be defined as

(8) SSBu=∑i=Pre,ProNi∑j=1NifijuNi−∑i=Pre,Pro∑j=1Nifiju∑i=Pre,ProNi2,SSWu=∑i=Pre,Pro ∑j=1Nifiju−∑i=Pre,Pro∑j=1Nifiju∑i=Pre,ProNi2,

where *f*_*ij*_(*u*) denotes frequency of the *u*th feature of the *j*th sample in the *i*th group (*i* = Pre or Pro). *N*^*i*^ denotes number of samples in the *i*th group (*i* = Pre or Pro). SS_*B*_(*u*) and SS_*W*_(*u*) are called sum of squares between groups and sum of squares within groups, respectively. If the sample means within groups are close to each other, SS_*B*_(*u*) will be small. If the sample means are close between two groups, SS_*W*_(*u*) will be small. Then the sample variance between groups *s*_*B*_^2^(*u*) and sample variance within groups *s*_*W*_^2^(*u*) can be given by

(9) sB2u=SSBudfB,sW2u=SSWudfW,

where d*f*_*B*_ and d*f*_*W*_ are called degrees of freedom in statistics. In this study, d*f*_*B*_ = 1 and d*f*_*W*_ = *N*^Pre^ + *N*^Pro^ − 2 = 254, respectively.

According to the statistic theory, the ratio between *s*_*B*_^2^(*u*) and *s*_*W*_^2^(*u*) obeys *F* sampling distribution with d*f*_*B*_ and d*f*_*W*_ degrees of freedom under the null hypothesis. Thus, we used ratio *F*(*u*) to measure the contribution of each feature defined as follows:

(10) $$\begin{array}{c} F\left(\;u\;\right)=\frac{s_{B}^{2}\left(u\right)}{s_{W}^{2}\left(u\right)}. \end{array}$$

**Figure 1:**
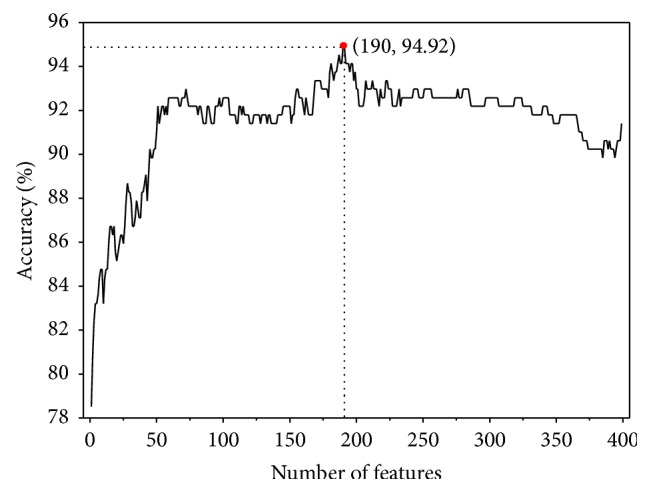
A plot to show the feature selection results. The maximum accuracy is 94.92% by using the top 190 features.

*F*(*u*) reveals how strong the *u*th feature is related to the group variables. Accordingly, the 400 dipeptides in (3) were ranked according to their *F*(*u*). Subsequently, the incremental feature selection (IFS) strategy was proposed to find an optimal of feature subset. In IFS procedure, we firstly examined the performance of the best feature with the highest *F*(*u*) by using cross-validation. Subsequently, a new feature with the second highest *F*(*u*) was added to form new feature subset which was also inputted into SVM and the accuracy was calculated. This process was repeated until 400 feature subsets were examined. By setting the number of features as abscissa and the Acc as ordinate, the IFS curves were plotted in Figure 1. From the figure, we observed that, in the jackknife cross-validation, the maximum Acc of 94.9% can be obtained by the top 190 features which are regarded as the optimal feature subset.

It is very important to compare the performance of different methods. However, it is not feasible because the benchmark datasets are different. Thus, we made a rough comparison and recorded the results in Table 1. Yang and Li proposed ID-based method to predict presynaptic and postsynaptic neurotoxins on a benchmark dataset with the sequence identity of <80% [5]. Thus, our method is superior to Yang's method. Song developed bilayer support vector machine to improve the accuracy [7]. We noticed that the sequence identity of the benchmark dataset reaches 90% which results in the overestimation of the method. Thus, our proposed model is more objective and real.

## 4. Conclusions

The knowledge for neurotoxin is conductive to the development of drug design and drug-target discovery. Thus, the aim of the study is to develop a computational method to predict presynaptic and postsynaptic neurotoxins. A new feature selection technique was proposed to optimize features and to improve prediction accuracy. The feature selection technique can also be used in other bioinformatics fields.

## Figures and Tables

**Table 1:** Comparison of prediction performance for presynaptic and postsynaptic neurotoxins.

	Sn	Sp	Acc
ID [5]	88.46	91.30	89.80
Bilayer SVM [7]	100.00	98.37	98.93
Our method	94.51	95.15	94.92
